# Randomized Controlled Trials of Technology-Based HIV/STI and Drug Abuse Preventive Interventions for African American and Hispanic Youth: Systematic Review

**DOI:** 10.2196/publichealth.7129

**Published:** 2017-12-13

**Authors:** David Córdova, Frania Mendoza Lua, Lauretta Ovadje, Ethan Hong, Berenice Castillo, Christopher P Salas-Wright

**Affiliations:** ^1^ School of Social Work University of Michigan Ann Arbor, MI United States; ^2^ College of Literature, Science, and the Arts University of Michigan Ann Arbor, MI United States; ^3^ School of Social Work Boston University Boston, MA United States

**Keywords:** technology, HIV, prevention, drug use, adolescents

## Abstract

**Background:**

HIV/sexually transmitted infections (STIs) and drug abuse remain significant public health concerns in the United States, and African American and Hispanic youth are disproportionately affected. Although technology-based interventions are efficacious in preventing and reducing HIV/STI and licit/illicit drug use behaviors, relatively little is known regarding the state of the science of these interventions among African American and Hispanic youth.

**Objective:**

The aim of this review is to identify and examine randomized controlled trials (RCTs) of technology-based HIV/STI and/or drug abuse preventive interventions for African American and Hispanic youth.

**Methods:**

We searched electronic databases (ie, PubMed, Proquest, PsycINFO, Ebscohost, Google Scholar) to identify studies between January 2006 and October 2016. RCTs of technology-based interventions targeting African American and Hispanic youth HIV/STI risk behaviors, including sexual risk, licit and illicit drug use, and HIV/STI testing were included.

**Results:**

Our search revealed a total of three studies that used an RCT design and included samples comprised of >50% African American and/or Hispanic youth. The follow-up assessments ranged from two weeks to six months and the number of participants in each trial ranged from 72 to 141. The three interventions were theory-driven, interactive, and tailored. The long-term effects of the interventions were mixed, and outcomes included reductions in sex partners, licit drug use, and condomless anal sex acts.

**Conclusions:**

Although technology-based interventions seem promising in the prevention of HIV/STI and drug abuse among African American and Hispanic youth, more research is needed.

## Introduction

HIV/sexually transmitted infections (STIs) and drug abuse remain major public health concerns in the United States. Although youth in general (defined here as adolescents 13-25 years of age) are at increased risk of HIV/STI, African American and Hispanic youth are disproportionately affected [1,2]. African American and Hispanic youth disproportionately engage in sexual risk and licit/illicit drug use behaviors [3,4], which increase their risk for STI and HIV infection [5,6]. However, HIV testing—a National Institutes of Health HIV/acquired immune deficiency syndrome (AIDS) research priority [7]—among African American and Hispanic youth remains underutilized [8]. Furthermore, many youths are not routinely screened for asymptomatic STIs, as recommended by the Centers for Disease Control and Prevention [2]. Youth experience multiple barriers to access HIV/STI preventive services, and exploring the use of technology as a platform to improve the delivery and uptake of such services is warranted.

Given the ubiquity of technology among youth, technology-based interventions provide an ideal platform for the dissemination of HIV/STI and drug abuse preventive interventions to populations disproportionately affected by HIV/STIs, including African American and Hispanic youth [6,9,10]. However, a gap in knowledge persists in the literature with respect to the state of the science of technology-based HIV/STI and drug abuse preventive interventions. The purpose of this review is to identify randomized controlled trials (RCTs) of technology-based HIV/STI and/or drug abuse preventive interventions for African American and Hispanic youth.

### The Prevalence of HIV/STI Risk Behaviors and HIV/STIs Among African American and Hispanic Youth

African American and Hispanic youth are disproportionately at risk of engaging in HIV/STI risk behaviors, including licit and illicit drug use and sexual risk behaviors [4,11-13]. Alcohol remains the most widely used licit drug among youth [4]. National surveillance data from the *Monitoring the Future* survey indicate that 9.5% of Hispanic 8th grade youth report past 30-day alcohol use, relative to 8.2% of non-Hispanic white and 6.9% of African American 8th grade youth, respectively [4]. By 10th grade, however, these trends change such that 23.0% of non-Hispanic white youth report past 30-day alcohol use, compared to 21.4% of Hispanic and 13.9% of African American youth, respectively [4]. Furthermore, 39% of non-Hispanic white youth report past 30-day alcohol use, compared to 34.9% of Hispanic and 21.8% of African American 12th grade youth, respectively [4]. Marijuana remains the most widely used illicit drug among youth [4]. Approximately 17.5% of Hispanic and 17.2% of African American 8th grade youth report lifetime marijuana use, compared to 10.0% of non-Hispanic white 8th grade youth, respectively [3]. Furthermore, 35.2% of Hispanic and 33.0% of African American 10th grade youth report lifetime marijuana use, relative to 28.9% of non-Hispanic white 10th grade youth, respectively [4]. Moreover, 50.3% of Hispanic 12th grade youth report lifetime marijuana use, relative to 43.6% of non-Hispanic white and 41.8% of African American 12th grade youth, respectively [4]. Licit and illicit drug use behaviors increase the risk of sexual risk behaviors, which in turn increase the vulnerability to STI and HIV infections [14]. National data from the *Youth Risk Behavior Surveillance* survey indicate that 44.4% Hispanic 9th-12th grade youth report condomless sex during last sexual intercourse, compared to 43.2% of non-Hispanic white youth [5]. Furthermore, 22.8% of Hispanic and 21.8% of African American 9th-12th grade youth report alcohol or drug use before last sexual intercourse, relative to 19.3% of non-Hispanic whites [5].

Due (in part) to the aforementioned risk behaviors, youth are at disproportionate risk of STI and HIV infections. Youth between the ages of 15 and 24 represent 25% of the sexually experienced population, and comprise nearly 26% and 50% of new HIV infections and new STIs, respectively [1,2]. African American and Hispanic youth are disproportionately affected by HIV/STIs. In 2014, an estimated 9731 youth were diagnosed with HIV in the United States; 78% of those diagnoses occurred in African American and Hispanic youth [3,5]. Indeed, the majority of these infections are among young men who have sex with men (YMSM). Importantly, 86% of young females acquire HIV through heterosexual sex [3,15,16]. Furthermore, African American and Hispanic female youth are disproportionately affected by STIs, which increase the risk of HIV infection [5].

### HIV and STI Testing Among African American and Hispanic Youth

Despite the disproportionate prevalence of HIV/STI risk behaviors and infections among African American and Hispanic youth, HIV testing remains low. Approximately 16.6% and 11.2% of African American and Hispanic 9th-12th grade youth report having ever been tested for HIV (not including tests when donating blood), respectively [3,5]. Although the low prevalence of HIV testing is alarming, even more disconcerting is the fact that between 2013 and 2015, HIV testing among youth has significantly decreased [5]. Concern for the downward trend in HIV testing among youth is underscored by recent shifts in the HIV/AIDS research priorities issued by the National Institutes of Health, in conjunction with practice recommendations issued by the US Preventive Services Task Force, which call for the urgent need for sound science and practice efforts aimed at improving HIV testing in youth [7,17,18].

Beyond the need to improve youth HIV testing in general, and in African American and Hispanic youth in particular, increasing STI testing remains a federal priority [7]. Screening for STIs is particularly important in preventing and reducing the transmission and acquisition of HIV, because the presence of an STI increases the risk of HIV infection [2]. Unfortunately, 60% of youth do not know they are infected with an STI, and many youths are only being tested based on their perceived risk and are not routinely screened for asymptomatic STIs [2,16]. However, this perceived risk is based, in part, on youth disclosing their HIV/STI risk behaviors to their clinician, and clinicians initiating patient-client risk communication. Researchers have shown that youth in clinical settings are not always forthcoming with respect to their HIV/STI risk behaviors, and some clinicians do not engage in youth-clinician HIV/STI risk communication [19]. Indeed, clinician-youth HIV/STI risk communication is brief or nonexistent, and some clinicians report discomfort or lack of HIV/STI risk communication training, which may partially explain the low rates of HIV and STI testing among African American and Hispanic youth [20,21].

### Harnessing the Power of Technology in the Prevention of HIV/STIs and Drug Abuse Among African American and Hispanic Youth

Technology is ubiquitous in the lives of many youths in the United States. For example, among youth aged 13-17 years, 73% and 58% have access to a smartphone or tablet, respectively [22]. Relative to non-Hispanic whites, African American youth are more likely to report smartphone ownership [22]. Thanks to the prevalence of these mobile devices, many youths utilize mobile technology to access the Internet and download apps. In fact, 74% of youth 12-17 years of age report accessing the Internet via mobile devices, and 58% report downloading apps to their smartphone or tablet [22].

Given the widespread use of mobile technology, it should not be surprising that mobile health (mHealth) interventions—the science and practice of health promotion through technology—are burgeoning [23]. In fact, data indicate that between 2010 and 2015 the overall app growth was 38.1% compounded annually over the past five years, compared to 41.9% for health-related apps [24]. In 2015, the number of health-related apps surpassed 165,000 [25]. Among adult populations, national data indicate that 36% of smartphone or tablet owners report having health-related apps on their devices [26]. Of those adults with apps, 60% report the usefulness of mHealth apps in achieving health behavior goals, 35% report helpfulness for medical care decision-making, and 38% report usefulness in asking their clinician new questions or seeking a second opinion [26]. However, limited data are available among youth populations. Extending the findings from adult populations, technology-based interventions may reduce some of the challenges that youth face that keep them from participating in face-to-face interventions (eg, transportation), offer a more cost-effective option, improve and extend the quality of care, and customize and prioritize the needs and preferences of youth [26,27]. In fact, HIV and drug abuse prevention scientists suggest that technology-based interventions show promise in preventing and reducing HIV and drug abuse among youth populations, but much work remains [28,29]. However, the literature on technology-based HIV/STI and drug abuse preventive interventions among youth remains limited, and even less is known about racial and ethnic minority populations.

A systematic review that focuses on African American and Hispanic youth populations is important for several reasons. First, African American and Hispanic youth are at disproportionate risk of engaging in HIV/STI risk behaviors (including licit and illicit drug use and sexual risk behaviors), acquiring STI and HIV infections, and HIV/STI testing is underutilized in these populations. Therefore, the identification of rigorously tested, culturally congruent, preventive interventions is warranted. Second, African American and Hispanic populations encompass important biological, psychological, social, historical, political, and cultural heterogeneity that underscores the need to develop and test culturally congruent preventive interventions [30-32]. In fact, researchers have shown how culturally specific preventive interventions may lead to optimal behavioral outcomes among African American and Hispanic youth [33,34]. Third, researchers conducting intervention research with African American and Hispanic youth populations have employed inclusion criteria with predominately African American or Hispanic samples in the identification of culturally congruent, efficacious preventive interventions [35,36]. Despite decades of prevention and intervention research, a review of RCTs comprised of primarily African American or Hispanic youth samples (ie, >70%) identified only 10 face-to-face interventions [37]. Unfortunately, the literature on technology-based interventions that are focused on African American and Hispanic youth is relatively limited. Therefore, the purpose of this review is to identify and examine RCTs of technology-based HIV/STI and/or drug abuse preventive interventions among African American and Hispanic youth.

## Methods

### Data Sources and Search Criteria

In the present review, we employed the methodological guidelines established for conducing systematic reviews [38-40], including the search, retrieval, selection, and coding of published articles. We searched electronic databases for relevant citations between January 2006 and October 2016, including PubMed, Proquest, PsycINFO, Ebscohost, and Google Scholar. Search terms included: “Intervention,” “Prevention,” “mHealth,” “eHealth,” “Telemedicine,” “HIV,” “STD (Sexually Transmitted Disease),” “STI,” “Risk Behavior,” “Substance Use,” “Substance Abuse,” “Adolescent,” “Web,” “Internet,” “Online,” “Mobile,” and “Phone.”

### Study Selection

For the purposes of this review, we included articles if they reported on preventive interventions delivered through mobile technologies or Web-based platforms that targeted HIV/STI risk behaviors (ie, sexual risk, and licit and illicit drug use), and HIV or STI testing. Additionally, studies had to employ an RCT design and focus on youth 13-25 years of age. Following the methodological approach employed by Szapocznik et al [37], we initiated the present review of RCTs with samples comprised of primarily African American or Hispanic youth (ie, >70%). This approach resulted in only one study that met the criteria. Therefore, the inclusion criteria in the present review was revised to a>50% threshold. We excluded articles that were nonrandomized trials, narrative reviews, observational studies, qualitative studies, treatment approaches (eg, medication adherence), face-to-face only interventions, did not include behavioral outcomes (eg, intentions, beliefs, knowledge), or adult populations. Additionally, we reviewed the references of the potential articles identified to ensure that any studies that may meet inclusion criteria of the present review were included.

**Figure 1:**
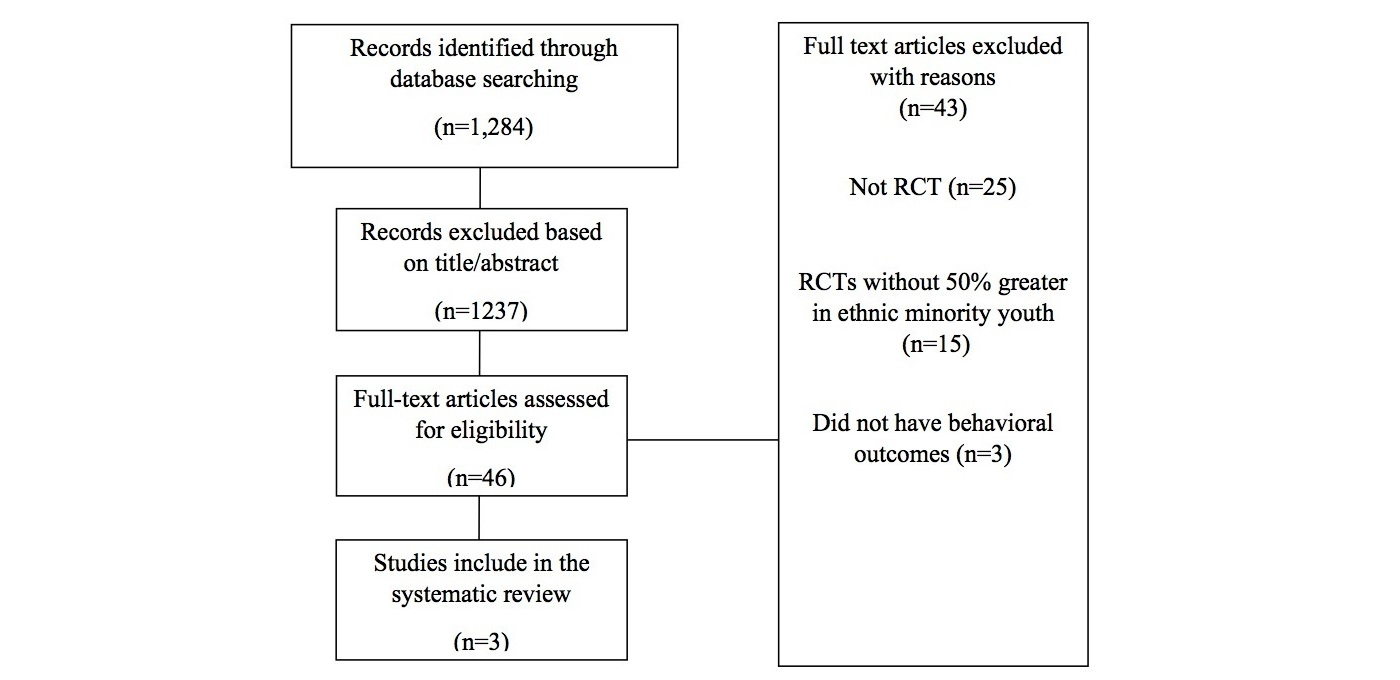
Primsa flow diagram.

Following the methodological approach for conducting systematic reviews [38-40], we screened and excluded articles that did not meet inclusion criteria (eg, nonrandomized trials, intervention was not technology-based, study did not include behavioral outcomes). Once studies were excluded that did not meet inclusion criteria, the full text of the remaining studies was reviewed and three were included for the purposes of this review (Figure 1).

### Data Extraction and Interrater Reliability

We conducted an interrater reliability check. Three of the authors (DC, LO, FML) independently screened and coded the articles that were identified as having fit the search criteria. Results were compared and any discrepancies regarding which articles met inclusion criteria were discussed until consensus was reached. We extracted the following information into a spreadsheet database: method of delivery (eg, mobile application, Web-based); author; publication year; study title; targeted sample; sample size; study site; study phase (eg, efficacy, effectiveness); and study findings.

## Results

As shown in Table 1, our search revealed three RCTs, all of which were published in 2013 or later. The studies examined the preliminary efficacy [41], efficacy [42], and comparative effectiveness [43] of a preventive intervention. Two studies used an intention-to-treat approach [41,43], one study used an attention control condition [42], and type of randomization was documented in two of the studies [41,42]. Sample sizes ranged from 72 [42] to 141 participants [43]. The longitudinal designs ranged from two weeks [43] to six months [42] follow-up, and two trials were registered with ClinicalTrials.gov [41,43]. The primary outcomes across the three studies included reductions in past 30-day sex partners [43], number of cigarettes smoked in the past 30 days [42], and rate of condomless anal sex acts [41].

Marsch et al [43] examined the comparative effectiveness of an interactive, customizable, Web-based intervention on sexual risk behaviors and the potential theoretical underpinnings by which change occurs (eg, skills acquisition), relative to a traditional educator-delivered intervention, among youth in an outpatient community-based substance abuse treatment clinic. Employing an intention-to-treat approach, 141 youth were randomized to either the experimental or control condition, and assessed at baseline and two-weeks postintervention.

**Table 1:** Randomized controlled trials of HIV/STI and/or drug abuse preventive interventions with African American and Hispanic youth.

Author	Platform	Sample	n	Study Stage	Study Design	Results
Marsch et al, 2015	Internet (Web)	Youth aged 12-18 years; 51% Hispanic, 43% Black	141	Effectiveness	RCT; face-to-face intervention or Web-delivered therapeutic education system. Follow-up conducted two weeks postbaseline.	Participants in both conditions reported reductions in past 30-day sex partners at two-week follow-up
Mason et al, 2015	Mobile (texting)	Youth aged 14-18 years; 90.8% African American	72	Efficacy	RCT; personalized motivational text messages or general health text control condition. Follow-up conducted at one, three, and six months postbaseline.	Participants in the experimental group had decreased past 30-day cigarette smoking, compared to control at six-month follow-up
Mustanski et al, 2013	Internet (Web)	Youth aged 18-24 years; 47% Hispanic, 12.7% African American	102	Preliminary efficacy	RCT; experimental condition or an online didactic HIV knowledge control condition. Follow-up conducted at six and twelve weeks.	Participants in the experimental condition reported a 44 % lower rate of unprotected anal sex acts at the 12-week follow-up

Marsch et al [43] provide descriptive statistics whereby participants could indicate both their race and ethnicity, with 44.6% identifying as Black and 51.0% Hispanic, respectively. Participants in the experimental condition received a self-directed, interactive, Web-based intervention. Guided by a community reinforcement approach to behavior therapy [44], the Web-based intervention aims to prevent and reduce HIV risk behaviors by improving skills, including decision-making and negotiation skills. The Web-based intervention is comprised of a computerized risk behavior assessment, followed by a total of 26 tailored modules. Using a computer at the clinic, the intervention was delivered in 60-minute sessions and the time for completing each module ranged between 10 and 30 minutes. On average, participants completed the sessions in approximately three sessions. Participants assigned to the control condition received two group or individual sessions, each of which lasted approximately one hour. Based on the National Institute on Drug Abuse HIV prevention principles in drug-using populations, the sessions focused on the epidemiology of HIV, promotive strategies to prevent and reduce HIV risk behaviors (including sexual risk and drug use), and HIV testing information [45]. Additionally, participants received a 20-minute youth-centered HIV prevention video. Findings indicated that participants in both conditions reported reductions in past 30-day sex partners (*P*=.05), however no significant differences in condition x time were observed. Furthermore, findings suggested that participants in both conditions reported improvements in condom use skills (*P*<.001), and this difference did not significantly differ across conditions two-weeks postintervention [43].

Mason et al [42] examined the efficacy of a text-based motivational interviewing intervention, relative to an attention control condition, on reducing smoking among a sample of predominately African American (90.8%) youth. Using a respondent-driven sampling design, 72 youth were randomized to either the experimental or attention control condition via block randomization, and were assessed at baseline, 1, 3, and 6-months postbaseline. Participants in the experimental condition received a text-based intervention that delivered 30 interactive text messages and three booster text messages over a five-day period that focused on rapport building, tobacco use feedback, social network information and feedback, and summary and plans with respect to the prevention of tobacco use [42]. By integrating motivational interviewing and social network counseling, the intervention aims to prevent and reduce tobacco use by increasing psychological precursors of behavior change, and reducing risk in close peer social networks. The attention control condition consisted of 30 health-based texts, including diet, exercise, and study habits. Findings indicated that participants in the experimental condition reduced the number of cigarettes smoked in the past 30 days, relative to the attention control condition. The results yielded a large and significant effect size, *F* (1,55)=4.39, *P*<.01, *η*^2^=0.17 [42]. Furthermore, youth in both conditions increased their readiness to change and willingness to refuse cigarettes from their friends, and decreased the number of close friends who were daily smokers, however no significant differences across conditions were found [42].

Mustanski et al [41] examined the preliminary efficacy of the *Keep It Up!* intervention, compared to an online didactic HIV knowledge condition. Using an intention-to-treat approach, 102 predominately African American (12.7%) and Hispanic (47%) YMSM were randomized via a computerized algorithm stratified by race, and assessed at baseline, immediate postintervention, prior to a 6-week booster session, and 12 weeks postintervention [41]. Participants in the experimental condition received the *Keep It Up!* intervention. Informed by the information motivation behavioral skills model of HIV risk behavior change, *Keep It Up!* consists of 7 tailored, interactive modules delivered across three sessions and completed at least 24 hours apart. *Keep It Up!* aims to prevent and reduce condomless sex via improvements in HIV knowledge, safer-sex self-efficacy, beliefs and intentions to use condoms, and condom use errors.

**Table 2:** Cochrane summary of findings for randomized controlled trials of HIV/STI and/or drug abuse preventive interventions with African American and Hispanic youth.

Author	Outcomes	Sample	Number of participants followed-up (studies)	Quality of the evidence (grade)	Anticipated absolute effects	
					Risk with control	Risk difference with technology intervention
Marsch et al, 2015	Reduction of 30-day sex partners	Youth aged 12-18 years; 51% Hispanic 43% African American	74 (1 RCT)	High	The mean reduction of 30-day sex partners was 0	Mean difference 5.67 higher (5.2 higher to 6.14 higher)
Mason et al, 2015	Number of cigarettes smoked in the past 30 days	Youth aged 14-18 years; 90.8% African American	72 (1 RCT)	High	The mean number of cigarettes smoked in the past 30 days was 0	Mean difference 0.6 lower (1.45 lower to 0.25 higher)
Mustanski et al, 2013	Condomless anal sex acts	Youth aged 18-24 years; 47% Hispanic, 12.7% African American	102 (1 RCT)	High	The mean condomless anal sex acts was 0	Mean difference 2.5 lower (6.19 lower to 1.19 higher)

Participants in the control condition received an HIV knowledge intervention consisting of 7 nontailored, noninteractive modules delivered across three sessions. The didactic intervention included HIV information and facts, including condom use, and the epidemiology of HIV and STIs in YMSM. Findings indicate that, relative to the control condition, participants in the experimental condition reported a 44% lower rate of unprotected anal sex acts at the 12-week follow-up (Risk Ratio=.056, *P*<0.05). Participants in both conditions showed a large increase in HIV knowledge at postintervention (Cohen’s d for *Keep It Up!*=0.75; control=0.87), however no significant differences were found across conditions [41]. As shown in Table 2, we computed Cochrane summary of findings to designate the score for each criterion in each article reviewed. All three articles that were reviewed received a high grade with respect to the quality of the evidence.

## Discussion

The present systematic review indicates that although advances in technology-based HIV/STI and drug abuse preventive interventions for African American and Hispanic youth have been made, only three studies that utilized an RCT design and included licit and illicit drug use or sexual risk behavior outcomes were identified. To the best of our knowledge, this is among the first systematic reviews focused on the state of the science on technology-based HIV/STI and drug abuse preventive interventions for African American and Hispanic youth. From this review, we identified four important gaps in the literature. First, there remains the need to employ methodological and statistical rigor, including the use or RCTs, to develop a fuller understanding of the effects of technology-based interventions on HIV/STI risk behaviors. Second, studies examining the long-term effects of technology-based HIV/STI and drug abuse preventive interventions are limited. Third, the identification of how, and for whom, technology-based prevention interventions do and do not work are needed. Finally, a dearth of rigorously tested technology-based HIV/STI and drug abuse preventive interventions for African American and Hispanic youth exist. Addressing these important gaps in the literature may be helpful in moving the field forward.

There remains a need to employ rigorous methodological and statistical analytic approaches to test the effects of technology-based HIV/STI and drug abuse preventive interventions. The RCT remains the standard when examining the impact of interventions [46], yet we identified only three studies that employed an RCT design. Therefore, future research should use RCT designs when examining the effects of technology-based interventions on HIV/STI and drug use among African American and Hispanic youth. Although studies examining the effects of technology-based HIV/STI and drug abuse preventive interventions using RCT designs in the United States are limited, perhaps we can draw from international studies aimed at moving the field forward. In fact, during the review process, we identified five studies conducted internationally that have employed an RCT design when examining HIV/STI risk behavioral outcomes [47-51]. For example, Ybarra et al [47] examined the effects of CyberSenga, a five-hour online healthy sexuality program on past 90-day condom use and abstinence over a six-month period. A total of 366 youth aged 12 years and older from Mbarara, Uganda, were randomized via parallel group with adaptive randomization to CyberSenga, CyberSenga with booster sessions, or a control group [47]. Although no statistically significant results were found among the main outcomes, study findings may help inform the development and testing of future technology-based interventions. For example, it seems that abstinent youth in the CyberSenga group benefited most in the short-term, whereas sexually active participants in the CyberSenga with booster group benefited long-term [47]. Future research to examine whether (and extent to which) these findings hold true in other youth populations is needed.

The long-term effects of technology-based HIV/STI and drug abuse preventive interventions are not well understood. Of the three studies included in this review, a relatively short-term follow-up assessment (ie, range from 2 weeks to 6 months) was used. Therefore, developing a more complete understanding of the long-term effects of technology-based HIV/STI and drug abuse preventive interventions on behavioral outcomes is essential to moving the field forward. Importantly, variations in the types of drug use behaviors and vulnerability to HIV infection exist. For example, alcohol use behaviors alone are not a risk factor for HIV infection. However, alcohol use behaviors increase the risk of engaging in sexual risk behaviors, which in turn enhances vulnerability to HIV infection [52]. Injection drug use (IDU), conversely, is a direct and efficient mechanism for the transmission and acquisition of HIV [53]. Despite this knowledge, we did not identify one single study with IDU as a behavioral outcome. Given the recent rise in opioid use in the United States and links to HIV infections [54], the development and testing of technology-based interventions focused on opioid use behaviors may be warranted.

Beyond the need to establish the long-term efficacy of technology-based interventions, there also remains the need to understand the pathways through which such interventions work. Indeed, scientists have underscored the necessity of developing and testing theory-driven interventions, which can aid in identifying the mechanisms through which preventive interventions have an impact on HIV/STI testing and risk behaviors among youth. Although our review suggests that investigators have developed theory-driven technology-based preventive interventions, the testing of the theoretical underpinnings that guide these interventions is limited. In fact, our review did not reveal a single study that tested for mediation. Therefore, future research should examine the pathways through which technology-based interventions may have an effect on HIV/STI risk behaviors and testing.

There also remains a need to develop a more complete understanding of the populations for which preventive interventions do and do not work [55,56]. That is, investigators examining both malleable and nonmalleable sample and contextual study characteristics may have great utility in working toward optimally efficacious technology-based interventions. For example, some studies indicate that the effects of technology-based interventions varied as a function of gender, suggesting a potential need for gender-specific interventions [48,51,57,58].

Although stark racial and ethnic disparities exist in the incidence of new STI and HIV infections among youth [1,2], our review revealed that the majority of studies did not include samples comprised of >50% racial and ethnic minority populations. Given the facts that African American and Hispanic youth disproportionately engage in HIV/STI risk behaviors [4,5], are disproportionately affected by STI and HIV infections [1,2], and underutilize HIV and STI testing [5], more research on the development and testing of technology-based HIV/STI interventions focusing on African American and Hispanic youth is warranted. Moreover, because African Americans (12%) and Hispanics (13%) are more likely to report being smartphone-dependent compared to non-Hispanic whites (4%) [22], mobile-technology interventions utilizing smartphones may be an effective approach to engaging and retaining African American and Hispanic youth in prevention services.

Findings from the present review may have implications for practice in at least two ways. First, improving clinician-delivered HIV/STI preventive services, including screening for HIV/STI risk behaviors and status, remains a federal priority [18]; however, clinicians face numerous challenges, including interpersonal and structural-level factors. Technology-based interventions have the potential to overcome many of the challenges associated with providing HIV/STI preventive services, including improving youth-clinician HIV/STI risk communication [27], providing access to HIV/STI prevention programs [28], and supporting and enhancing traditional prevention models by supplementing face-to-face intervention efforts [59]. Second, technology-based interventions have the potential to improve clinicians’ delivery of targeted, tailored, and cost-effective HIV/STI preventive services. For example, some technology-based interventions include a youth risk assessment [43,60], which can be used to deliver targeted and tailored HIV/STI prevention services based on the youth’s specific risk behaviors.

Findings from the present review should be interpreted in light of an important limitation. Specifically, we did not include quasi-experimental studies in our review. RCTs may not always be a feasible intervention design, and well-designed quasi-experimental studies may have added to the evidence-base of the present review. We did not include quasi-experimental studies in our review for several reasons. First, the key distinction between quasi-experimental studies and RCTs is the random assignment of participants to either the treatment or control group. This distinction is particularly important during analyses given the increased potential of nonequivalence between groups, yielding challenges in comparisons. Second, the aim of the present review is to establish the efficacy of technology-based HIV/STI and drug abuse preventive interventions for African American and/or Hispanic youth. As such, it was pertinent that our review focus on RCTs, which has been established as the primary method that allows for better causation implications. Finally, in our systematic review, we followed the methodological approach of including only RCTs, as employed in other studies in medical Internet research [61,62].

### Conclusions

In summary, although important advances have been made in the development and testing of technology-based HIV/STI and drug abuse preventive interventions, few interventions that target *both* sexual risk and licit/illicit drug use behaviors—behaviors that often co-occur [5]—and even fewer interventions aimed at improving HIV and STI testing among youth exist [27]. Importantly, efficacious interventions for African American and Hispanic youth are limited. The results from this review demonstrate the promise of technology-based interventions to prevent and reduce new STI and HIV infections among African American and Hispanic youth. Although there is some empirical support for technology-based HIV/STI preventive interventions, much work remains. The present review identified four critical gaps, including the need to develop a more complete understanding of: (1) the effects of technology-based interventions on HIV/STI risk behaviors and testing through cutting-edge methodological and statistical approaches, including the use or RCTs; (2) the long-term effects of technology-based interventions; (3) the pathways through which such interventions work; and (4) for whom these interventions do and do not work. For example, there have been a relatively large number of studies focused on establishing the feasibility and acceptability of technology-based interventions, compared to studies focused on demonstrating the short-term and long-term behavioral outcomes [63]. Indeed, findings from this review suggest that there remains a need for the development and testing of technology-based interventions for populations most affected by HIV/STIs, including African American and Hispanic youth. This review will help advance the state of the science on technology-based youth HIV/STI and drug abuse preventive interventions.

## Abbreviations

IDU: injection drug use
mHealth: mobile health
RCT: randomized controlled trial
STI: sexually transmitted infection
YMSM: young men who have sex with men
